# Differential item functioning analysis on the Geriatric Depression Scale-15: An iterative hybrid ordinal logistic regression

**DOI:** 10.37796/2211-8039.1098

**Published:** 2021-12-01

**Authors:** Elahe Allahyari

**Affiliations:** Department of Epidemiology and Biostatistics, School of Health, Social Determinants of Health Research Center, Birjand University of Medical Sciences, Birjand, Iran

**Keywords:** The Geriatric Depression Scale, Depression, Differential item functioning (DIF), Ethnicity/Race, Home bound

## Abstract

The elderly population has extensively increased globally, so depression like a common problem in late life may convert to one of the economic, social, and health challenges of the 21st century. Due to the high cost of clinical diagnosis of depression, it is necessary to provide effective questionnaires like the 15-item Geriatric Depression Scale (GDS-15) for screening. But, the measurement invariance of GDS-15 is still unknown in the general population. In our study, 1473 participants of all Iran’s ethnic groups were asked to answer GDS-15 and demographic factors such as human settlements, employment, disease, marital status, age, gender, homebound, financial status, and ethnicity. Then, the lordif package in R 3.1.3 was used to assess differential item functioning (DIF) items that behave unevenly across demographic factors. Our findings reveal that women, homebound patients, poorer, and non-Persian mother tongue score classic psychological symptoms higher than peoples of the same depression score in other groups. Since, psychologists have to remove or replace these items before using this questionnaire for screening geriatric depression.

## 1. Introduction

In late life, depression is one of the reversible disorders that increased healthcare expenses and decreased quality of life [1]. In 1999, a systematic review reported only 13.5% of the depression prevalence in elderly people aged 55 and older, but depressive symptoms had proliferated over time [2, 3]. Since population dynamic is one of the most important factors to determine health care needed in society, the growing trend of the elderly population can convert depression into one of the economic, social, and health challenges of the 21st century [4, 5]. To prevent this problem, we should investigate methods for prompt recognition of this illness. The clinical interview is time-consuming and costly, while screening and diagnostic instruments are available to overcome these dilemmas [6].

When assessing geriatric depression, screening instruments should comprise simple and easily understood items to fit this population. Furthermore, psychological symptoms should have greater weight than somatic ones because of their adequate power to discriminate depressed from non-depressed elderly [7]. The 15-item Geriatric Depression Scale (GDS-15), which has 92% sensitivity and 89% specificity, is one of the most popular instruments to meet these expectations [7, 8]. It also had acceptable validity and reliability in clinical practice and research [9–11].

But before assessing group differences on a questionnaire, test items should be fair and appropriate for assessing the knowledge in a specific area across different groups of examinees [12]. In this condition, the differences in performance between groups reflect true differences in the ability level, and there are not due to some items that do not behave comparably for subjects from different groups. Differential item functioning (DIF) signals, that factors related to group membership, affect the probability of response thus threaten fair assessment [13, 14]. So, DIF analysis is one important part of assessing validity, especially in psychological instruments [15, 16].

## 2. Purpose

Kim, et al. used Item Response Theory (IRT) to discover DIF items in Beck Depression Inventory (BDI) [17]. They showed that persons with low and high depression answered cognitive and somatic symptom items differently. Two other studies reveal that GDS-15 had no significant DIF among workers and health care patients for age, level of education, sex, and race(18, 19). But I cannot find DIF analyses of GDS-15 in the general population or other demographic variables marital status, ethnicity, human settlements, etc. So, this study focused on whether test takers have similar knowledge perform on individual test items of GDS-15 regardless of their age, marital status, ethnicity, human settlements, gender, homebound, chronic illness, employment, and financial status.

## 3. Methods

### 3.1. Statistical analysis

In the logistic regression model (OLR), the logit of observing each dichotomous item response relates to two explanatory variables: observed ability (θ), a continuous variable, and a category group variable (G) as follows:


(1) $$\text{logit }(\text{p}(\text{Y}=1|\text{x}))=\beta_{0}+\beta_{1}\theta +\beta_{2}\text{G}+\beta_{3}\theta \text{G}$$

Under this formulation, an item shows uniform DIF if β_2_ ≠ 0 and β_3_ = 0, and non-uniform DIF if β_3_ ≠ 0. And these hypotheses can be tested using the G^2^ likelihood ratio statistic [20]. To access the size of DIF, effect size measures of Δβ_1_^1^ and ΔR_1_^2^ were estimated in uniform DIF items and ΔR_2_^3^ was estimated in non-uniform DIF items [21, 22]. In this article, Δβ_1_, ΔR_1_, and ΔR_2_ were assessed largely when were higher than 0.01, 0.07, and 0.07, respectively [23, 24].

If the test contains biased items, then a biased criterion of the ability will be used for investigating DIF in the OLR method [25, 26]. But, if the latent variable of IRT models were substituted at the ability of OLR models, the logistic regression analysis could overlook this potential limit of ability parameters on DIF detection. To meet this, the lordif package in R 3.1.3 detected DIF items by OLR methods in the first step. Then, incorporate IRT-derived ability estimates rather than the ability parameter in the OLR model only for each group category of DIF items separately. And, it detected DIF items by the OLR method again. The two last steps were repeated until reaching the same DIF items in two consecutive stages [27]. Finally, the effect of removing uniform DIF items was also assessed on the group differences.

### 3.2. Measures

In 1983, a team of clinicians and psychiatry researchers picked out 100 of the most efficient items that would not alarm patients or make them overly defensive. Items also incorporated unique elderly cognitive complaints and had a yes/no format to make a simpler self-rating scale for the patients. Then, 47 participants from elderly depressives and normals in California were asked to answer questions. And, the best-correlated items with depressive symptoms shaped the 30-item Geriatric Depression Scale (GDS) [7]. In 1986, practical items shaped the GDS short form with 15 items [28]. Except for items 1, 5, 7, 11, and 13, which scored negatively, positive answers indicated depression. Depression score suggested normal person (range 0–4), mild (range 5–8), moderate (range 9–11), or severe (range 12–15) depression. To ensure the accuracy of the translation, independent translators converted all scale items to Persian and back to English in 2005. Then, 204 elderly people were asked to answer the questionnaire according they are felt over the past week. Then, the test-retest reliability of the scale was assessed. The Persian version had acceptable reliability and validity (test-retest reliability = 0.58, Cronbach’s α = 0.9, r_split-half_ = 0.89) as original one (test-retest reliability=0.64, Cronbach’s α = 0.81) [29, 30].

### 3.3. Participants

However, the official language in Iran is Persian; about half of the Iranian population had not the Persian mother tongue. This paper was also intended to consider the impact of this factor on the person’s perception of the Persian version of GDS-15. So, we randomly selected 800 people aged 60 and older in both Persian and non-Persian mother tongue. In choosing people who had not Persian mother tongue tried to consider Iranian ethnicity’s proportions [31]. From April to October 2017, information was gathered from different 16 cities (Isfahan, Nain, Tehran, Birjand, Dihook, Shahekord, Mashhad, Tabriz, Fereidan county, Sanandaj, Kermanshah, Lordegan, Khoramabad, Zirkooh county, Ahwaz, Agh-Ghala). Study persons had not experienced chronic sorrow during the past month and completed informed consent. The incomplete questionnaires were removed from the study (116 for Persian mother tongue, 11 for non-Persian mother tongue). Finally, the lordif and psych packages analyzed information of 684 Persian, 297 Turks, 96 Lurs, 100 Kurds, 98 Baluchis, 98 Arab, and 100 Turkmens.

## 4. Results

Item responses were available from 1473 participants (684 Persian mother tongue and 789 non-Persian mother tongue). The mean ages of the analysis population were 69.22 and 69.40 years old for males and females, respectively. Most of the respondents lived in the city (53.6%), have a chronic medical illness (70.8%), and were married (76.3%). About 16.2 percent had home health care, 20.2 percent were currently employed, with financial status 37.5% poor, 21% making ends meet, and 41.5% rich. As Table 1 clearly shows, Cronbach’s alpha and coefficient omega were in an acceptable range, but the Standardized Root Mean Squared Residuals (SRMR) were the only Confirmatory Factor Analysis (CFA) indices that were in recommended range by Hu and Bentler [32, 33]. For non-Persian mother tongues, even SRMR was out of range.

### 4.1. DIF analysis

In Tables 2 and 3, DIF analysis is provided across human settlements, disease, employment, marital status, age, gender, homebound, financial status, and ethnicity. The chi-square statistics declare 10, 5, 8, 4, 3, 11, 11, 14, and 5 DIF items across these factors, respectively. But, none of the items had large non-uniform DIF (ΔR_2_ ≤ 0.0189). In uniform DIF items, Δβ_1_ is often large, but the only item 4 in financial status shows large ΔR_1_ (ΔR_1_ = 0.0957). The CvBL was non-significant only in 13 of 60 uniform DIF items (items 1, 8, 9, and 15 for human settlement groups, item 4 for people with and without chronic illness, items 2, 4, 8 for employment groups, item 6 for gender groups, items 2 and 15 for homebound groups, item 9 for financial status groups, and item 15 for ethnic groups).

**Fig. 1:**
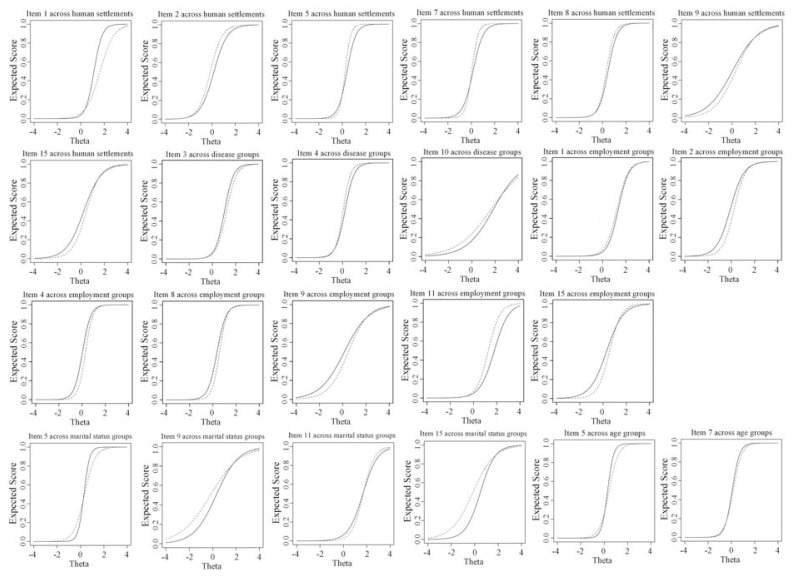
test characteristic curve of DIF items for human settlements (village (solid line), city (dashed line)), disease (without chronic illness (solid line), with chronic illness (dashed line)), employment status (no (solid line), yes (dashed line)), marital status (married (solid line), single (dashed line)), and age (young-old (solid line), old-old (dashed line)) groups according to the hybrid OLR/IRT.

Because there was more than one uniform DIF item in all study factors, item score functions would be helpful in the investigation of additive or cancel-out effect. To do that, dashed lines are used for city habitat, chronic illness patients, single, old-old people, and yes categories in Fig. 1. For human settlement, four uniform DIF items are in the opposite direction of other threes, and uniform DIF items can cancel out effect size at the domain level. Items 4 and 10 also canceled out item 3 across the presence or absence of chronic medical illness. About employment status, large effect sizes in items 1 and 11 cancel out not only non-significant uniform DIF in items 2, 4, and 8 but also significant uniform DIF in items 9 and 15. In marital status, items 5 and 11 cancel out each other, but items 9 and 15 go in one direction. Two uniform DIF items in age groups also go in one direction. But, if we focused on both effect size measures Δβ_1_ and ΔR_1_, additive effects would not be meaningful in marital status and age groups.

**Fig. 2:**
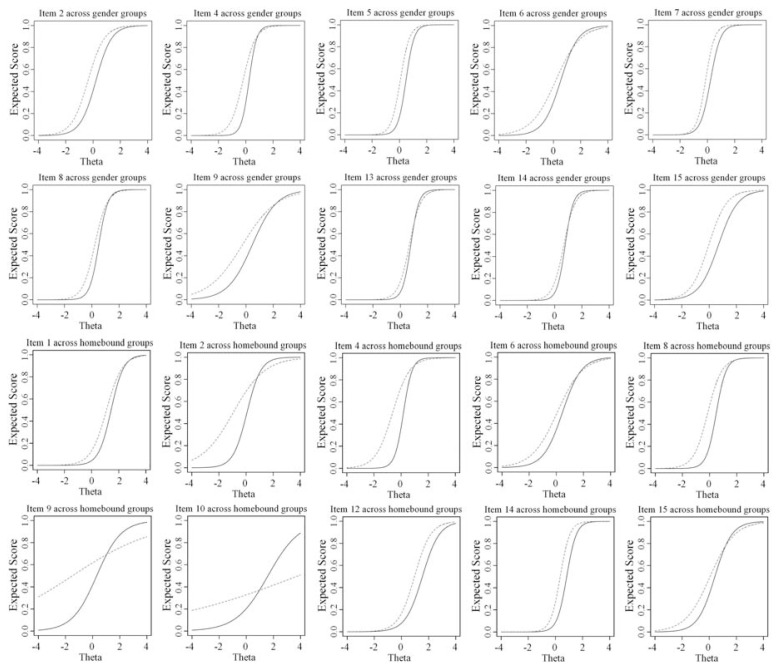
Test characteristic curve of DIF items for gender (male (solid line), female (dashed line)) and homebound (no (solid line), yes (dashed line)) groups according to the hybrid OLR/IRT.

**Fig. 3:**
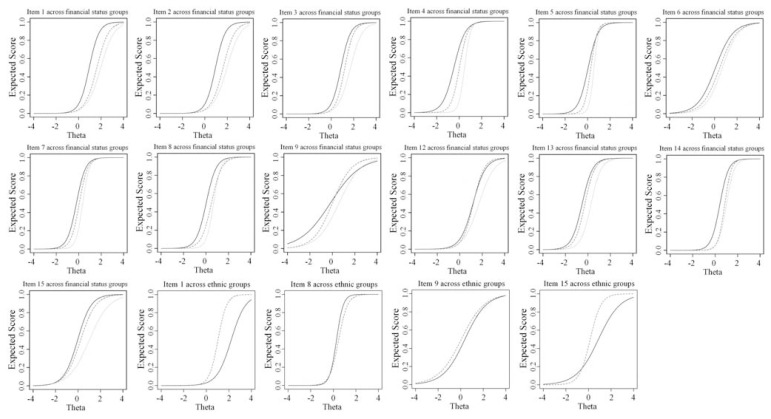
Test characteristic curve of DIF items for financial status (poor (solid line), make ends meet (dashed line), and rich (dotted line)) and ethnic (Persian (solid line), non-Persian (dashed line)) groups according to the hybrid OLR/IRT.

In Figs. 2 and 3, item score functions show gender, homebound, financial status, and ethnic groups. For gender, homebound, and financial status groups, all uniform DIF items go in one direction. Across ethnic groups, only item 8 is in the opposite direction by items 1, 9, and 15, so it cannot cancel out them. The observed additive effect causes female, homebound patient, poor person, and non-Persian mother tongue scores their depression higher.

Table 4 assesses the effect of removing uniform DIF items on group differences in studied factors. As the table clearly shows, the only group differences change the p-value of significant (<0.001) to non-significant (0.17 and 0.035 in gender and financial status) after removing uniform DIF items. However, like other study factors, removing DIF items does not change ethnic and employment status group difference (P-value<0.01). According to all presented findings, uniform DIF is more noticeable in items 5 and 15 for gender groups, items 4, 8, and 14 for homebound groups, items 1, 3, 4, 5, 7, 8, 13, 14, and 15 for financial status groups, and item 1 for ethnic groups.

## 5. Discussion

As we know, interpretation of GDS item scores across different group memberships was compared in four studies: Chinese pneumoconiosis workers, homebound patients in New York, a large longitudinal in old age Italian, and [18, 34, 35]. Across level of education and age groups, Rasch models show DIF in items 3, 4, 9, and 10 for silicosis workers in Hong Kong. But, the Persian version of GDS-15 had no DIF for variable age like the American version. Since Tang et al. studied a limited population, their findings could not be recommended in the general population like this study findings. About health care patients in Westchester, there is also a similar limitation. Hence, in contrast to health care women in America, Iranian women rated their depression higher than men in items 5 and 15. White Americans and other homebound patients also show DIF in item 10, while we evaluate clinically significant DIF in item 1 across the Persian race.

Living in underdeveloped regions can explain the reason why most of the no-Persian population unsatisfied with their life in Iran [36]. Furthermore, old women experience bad spirits and self-deficiency more than men of the same age, because less well-educated, widowhood and low incomes are more common among older women than older men. [37]. So, they score items “Are you in good spirits most of the time?” and “Do you think that most people are better off than you are?” higher than men.

Similar reasons maybe exist for DIF items in homebound and financial status. Rich people believe that they create their future, not other people or events [38]. So, rich people are in control of their lives and feel more satisfied, lively, excited, happy, strong, full of energy about themselves and their situation [39]. And rich people evaluate themselves lower in items 1, 3, 4, 5, 7, 8, 13, 14, and 15 of the GD depression scale. Also, social isolation and financial worries about medication can cause two important reasons why homebound elderlies score themselves bored, helpless, and even feeling their situation worse than others [40–42].

Our study results are more reliable than previous studies for two reasons. First, as it had been mentioned in before studies, data were drawn from the limited population (pneumoconiosis or homebound patients) while our study selected samples from all over Iran, so the generalizability of previous findings would come under suspicion [14, 18, 19]. Second, stringent assumptions of rash models might lead to erroneous conclusions that contrast the OLR models. Also, logistic models show unacceptable power, when ability parameter distribution is asymmetrical with an even sample size of 600 [43]. But using the iterative hybrid ordinal logistic regression/item response theory model for DIF detection gave us the strength to overcome these difficulties [26]. Finally, despite all the strengths in this study, further studies are recommended for detecting DIF between cultures and replacing or removing observed DIF items according to the psychologist’s idea.

However, The GDS-15 is useful for the detection of depression across human settlements, disease, employment status, marital status, and age groups. This tool might be misleading to compare depression between men and women, home healthcare patients and others, Persian and non-Persian mother tongues, and especially people of different levels of income. So, confirmation studies are warranted on the use of the shortened version of GDS in different ethnic, gender, and homebound groups. But, most of the items are needed to replace in different income levels.

## Figures and Tables

**Table 1:** The results of validity and reliability in each demographic factor category separately.

		Alpha^a^	Omega^b^	RMSEA^c^	SRMR^d^	CFI^e^	TLI^f^
Human settlements	Village	0.86	0.87	0.105^g^	0.077	0.781^g^	0.745^g^
	City	0.88	0.89	0.106^g^	0.076	0.814^g^	0.784^g^
Disease	With chronic illness	0.86	0.88	0.106^g^	0.078	0.789^g^	0.753^g^
	Without chronic illness	0.88	0.89	0.099^g^	0.066	0.830^g^	0.802^g^
Employment status	Yes	0.88	0.89	0.107^g^	0.072	0.796^g^	0.762^g^
	No	0.87	0.88	0.106^g^	0.077	0.791^g^	0.756^g^
Marital status	Married	0.86	0.88	0.103^g^	0.073	0.796^g^	0.762^g^
	Single	0.85	0.86	0.102^g^	0.079	0.775^g^	0.738^g^
Age	60–74	0.87	0.88	0.102^g^	0.072	0.806^g^	0.774^g^
	75–90	0.87	0.89	0.102^g^	0.077	0.807^g^	0.775^g^
Gender	Male	0.88	0.89	0.102^g^	0.067	0.820^g^	0.790^g^
	Female	0.85	0.87	0.103^g^	0.079	0.783^g^	0.747^g^
Homebound	Yes	0.81	0.84	0.082^g^	0.070	0.815^g^	0.785^g^
	No	0.87	0.88	0.104^g^	0.075	0.803^g^	0.770^g^
Financial status	Rich	0.86	0.87	0.105^g^	0.078	0.793^g^	0.758^g^
	Make ends meet	0.87	0.88	0.101^g^	0.075	0.801^g^	0.768^g^
	Poor	0.86	0.87	0.104^g^	0.075	0.782^g^	0.746^g^
Ethnic	Persian	0.87	0.88	0.102^g^	0.074	0.809^g^	0.777^g^
	Non-Persian	0.87	0.88	0.112^g^	0.082^g^	0.776^g^	0.739^g^
Total		0.87	0.88	0.102^g^	0.071	0.810^g^	0.778^g^

a Cronbach’s alpha coefficient.

b Coefficient omega.

c Root Mean Squared Error of Approximate (RMSEA).

d Standardized Root Mean Squared Residual (SRMR).

e Comparative Fit Index (CFI).

F Tucker-Lewis Index (TLI).

g Represent model fit indices out of acceptable range Hu and Bentler.

**Table 2:** The results of the hybrid OLR/IRT DIF analysis on the Geriatric Depression Scale (GDS-15) for human settlements, disease, employment status, marital status, age.

	Non-uniform		uniform		
		
	χ^2^(P)	ΔR^2^	χ^2^(P)	ΔR_1_	Δβ_1_
Human settlements					
1. Are you basically satisfied with your life?	15.138* (0.0000)	0.0161	7.879* (0.0050)	0.0065	0.0028
2. Have you dropped many of your activities and interests?	0.948 (0.3302)	0.0005	15.138* (0.0000)	0.0115	0.0344*
4. Do you often get bored?	15.138* (0.0000)	0.0083	0.820 (0.3651)	0.0004	0.0022
5. Are you in good spirits most of the time?	15.138* (0.0000)	0.0091	10.651* (0.0011)	0.0053	0.0112*
6. Are you afraid that something bad is going to happen to you?	12.532* (0.0004)	0.0063	0.299 (0.5845)	0.0002	0.0014
7. Do you feel happy most of the time?	15.138* (0.0000)	0.0093	7.643* (0.0057)	0.0037	0.0121*
8. Do you often feel helpless?	0.238 (0.6259)	0.0001	7.379* (0.0066)	0.0038	0.0087
9. Do you prefer to stay at home, rather than going out and doing new things?	1.111 (0.2918)	0.0005	5.717* (0.0168)	0.0028	0.0053
10. Do you feel you have more problems with memory than most?	13.831* (0.0002)	0.0084	3.480 (0.0621)	0.0021	0.0074
15. Do you think that most people are better off than you are?	2.665(0.1026)	0.0013	9.293* (0.0023)	0.0047	0.0010
Disease					
3. Do you feel that your life is empty?	0.041 (0.8388)	0.0000	4.388* (0.0362)	0.0033	0.0104*
4. Do you often get bored?	4.647* (0.0311)	0.0023	5.262* (0.0218)	0.0026	0.0034
6. Are you afraid that something bad is going to happen to you?	15.138* (0.0000)	0.0109	0.342 (0.5585)	0.0002	0.0039
9. Do you prefer to stay at home, rather than going out and doing new things?	6.617* (0.0101)	0.0033	2.813 (0.0935)	0.0014	0.0111
10. Do you feel you have more problems with memory than most?	1.050 (0.3054)	0.0006	6.498* (0.0108)	0.0040	0.0236*
Employment status					
1. Are you basically satisfied with your life?	0.768 (0.3807)	0.0006	3.858* (0.0495)	0.0032	0.0167*
2. Have you dropped many of your activities and interests?	1.739 (0.1873)	0.0009	10.651* (0.0011)	0.0053	0.0096
4. Do you often get bored?	0.758 (0.3839)	0.0004	12.532* (0.0004)	0.0063	0.0006
8. Do you often feel helpless?	1.636 (0.2009)	0.0008	9.000* (0.0027)	0.0046	0.0029
9. Do you prefer to stay at home, rather than going out and doing new things?	1.618 (0.2034)	0.0008	6.745* (0.0094)	0.0033	0.0165*
10. Do you feel you have more problems with memory than most?	7.247* (0.0071)	0.0044	0.133 (0.7158)	0.0001	0.0044
11. Do you think it is wonderful to be alive now?	0.688 (0.4067)	0.0006	7.953* (0.0048)	0.0075	0.0332*
15. Do you think that most people are better off than you are?	4.786* (0.0287)	0.0024	8.869* (0.0029)	0.0045	0.0120*
Marital Status					
5. Are you in good spirits most of the time?	15.138* (0.0000)	0.0179	7.809* (0.0052)	0.0039	0.0295*
9. Do you prefer to stay at home, rather than going out and doing new things?	3.049 (0.0808)	0.0015	5.010* (0.0252)	0.0025	0.0309*
11. Do you think it is wonderful to be alive now?	5.976* (0.0145)	0.0056	6.843* (0.0089)	0.0064	0.0551*
15. Do you think that most people are better off than you are?	6.635* (0.0100)	0.0033	15.138* (0.0000)	0.0109	0.0389*
Age					
5. Are you in good spirits most of the time?	15.138* (0.0000)	0.0086	9.293* (0.0023)	0.0047	0.0212*
7. Do you feel happy most of the time	1.322 (0.2503)	0.0006	5.087* (0.0241)	0.0025	0.0125*
15. Do you think that most people are better off than you are?	6.764* (0.0093)	0.0034	0.097 (0.7551)	0.0000	0.0018

Δβ_1_: Crane van Belle and Larson criterion or |[β_1_ (Model_without G_) - β_1_ (Model_with G_)]/β_1_ (Model_without G_)|.

ΔR_1_ = 1-ln[L(Model_with G_)]/ln[L(Model_without G_)]; ΔR_2_ = 1-ln[L(Model_with θG_)]/ln[L(Model_without θG_)].

* Represent the items showing uniform or non-uniform DIF;

P: p-value of Chi-square statistic; χ^2^: the value of the difference in −2 log-likelihood of models with and without group variable G for uniform DIF and models with and without interaction θG for nonuniform DIF.

**Table 3:** The results of the hybrid OLR/IRT DIF analysis on the Geriatric Depression Scale (GDS-15) for gender, homebound, subjective financial status, and ethnic.

	Non-uniform		uniform		
		
	χ^2^(P)	ΔR_2_	χ^2^(P)	ΔR_1_	Δβ_1_
Gender					
2. Have you dropped many of your activities and interests?	0.019 (0.8913)	0.0000	15.138* (0.0000)	0.0269	0.0310*
4. Do you often get bored?	15.138* (0.0000)	0.0125	15.138* (0.0000)	0.0261	0.0456*
5. Are you in good spirits most of the time?	0.460* (0.4976)	0.0002	15.138* (0.0000)	0.0353	0.0824*
6. Are you afraid that something bad is going to happen to you?	7.707* (0.0055)	0.0039	15.138* (0.0000)	0.0099	0.0095
7. Do you feel happy most of the time?	15.138* (0.0000)	0.0015	15.138* (0.0000)	0.0208	0.0367*
8. Do you often feel helpless?	5.749* (0.0165)	0.0029	15.138* (0.0000)	0.0145	0.0282*
9. Do you prefer to stay at home, rather than going out and doing new things?	5.814* (0.0159)	0.0029	15.138* (0.0000)	0.0176	0.0155*
10. Do you feel you have more problems with memory than most?	6.418* (0.0113)	0.0039	1.541 (0.2145)	0.0009	0.0001
13. Do you feel full of energy?	2.450 (0.1175)	0.0012	15.138* (0.0000)	0.0336	0.0484*
14. Do you feel that your situation is hopeless?	4.834* (0.0279)	0.0028	9.644* (0.0019)	0.0056	0.0188*
15. Do you think that most people are better off than you are?	0.994 (0.3188)	0.0005	15.138* (0.0000)	0.0329	0.0422*
Homebound					
1. Are you basically satisfied with your life?	1.864 (0.1722)	0.0015	15.137* (0.0001)	0.0126	0.0113*
2. Have you dropped many of your activities and interests?	15.138* (0.0000)	0.0119	15.138* (0.0000)	0.0104	0.0055
4. Do you often get bored?	15.138* (0.0000)	0.0105	15.138* (0.0000)	0.0272	0.0389*
5. Are you in good spirits most of the time?	12.116* (0.0005)	0.0061	0.516 (0.4727)	0.0003	0.0009
6. Are you afraid that something bad is going to happen to you?	2.326 (0.1272)	0.0012	6.386* (0.0115)	0.0032	0.0104*
8. Do you often feel helpless?	6.064 (0.0138)	0.0031	15.138* (0.0000)	0.0289	0.0452*
9. Do you prefer to stay at home, rather than going out and doing new things?	15.138* (0.0000)	0.0117	15.138* (0.0000)	0.0074	0.0225*
10. Do you feel you have more problems with memory than most?	15.138* (0.0000)	0.0104	4.076* (0.0435)	0.0025	0.0257*
12. Do you feel pretty worthless the way you are now?	0.402 (0.5262)	0.0003	15.138* (0.0000)	0.0151	0.0162*
14. Do you feel that your situation is hopeless?	0.204 (0.6512)	0.0001	15.138* (0.0000)	0.0262	0.0468*
15. Do you think that most people are better off than you are?	4.364* (0.0367)	0.0022	5.666 (0.0173)	0.0028	0.0084
Financial status					
1. Are you basically satisfied with your life?	4.636* (0.0313)	0.0057	15.138* (0.0000)	0.0593	0.0913*
2. Have you dropped many of your activities and interests?	7.774* (0.0053)	0.0051	15.138* (0.0000)	0.0336	0.0321*
3. Do you feel that your life is empty?	3.133 (0.0767)	0.0038	15.138* (0.0000)	0.0521	0.0955*
4. Do you often get bored?	15.138* (0.0000)	0.0151	15.138* (0.0000)	0.0957	0.1843*
5. Are you in good spirits most of the time?	15.138* (0.0000)	0.0189	15.138* (0.0000)	0.0466	0.1278*
6. Are you afraid that something bad is going to happen to you?	0.036 (0.8492)	0.0002	15.138* (0.0000)	0.0172	0.0131*
7. Do you feel happy most of the time?	2.997 (0.0834)	0.0024	15.138* (0.0000)	0.0370	0.0674*
8. Do you often feel helpless?	4.904* (0.0268)	0.0037	15.138* (0.0000)	0.0569	0.1264*
9. Do you prefer to stay at home, rather than going out and doing new things?	5.377* (0.0204)	0.0038	15.138* (0.0000)	0.0166	0.0064
10. Do you feel you have more problems with memory than most?	7.707* (0.0055)	0.0064	1.002 (0.3168)	0.0014	0.0017
12. Do you feel pretty worthless the way you are now?	3.571 (0.0588)	0.0043	10.206* (0.0014)	0.0099	0.0125*
13. Do you feel full of energy?	0.007 (0.9345)	0.0001	15.138* (0.0000)	0.0560	0.0702*
14. Do you feel that your situation is hopeless?	3.045 (0.0810)	0.0029	15.138* (0.0000)	0.0612	0.1698*
15. Do you think that most people are better off than you are?	7.406* (0.0065)	0.0051	15.138* (0.0000)	0.0427	0.0455*
Ethnic					
1. Are you basically satisfied with your life?	15.136* (0.0001)	0.0120	15.138* (0.0000)	0.0597	0.0736*
6. Are you afraid that something bad is going to happen to you?	7.325* (0.0068)	0.0037	1.121 (0.2896)	0.0006	0.0079
8. Do you often feel helpless?	5.492* (0.0191)	0.0028	15.137* (0.0001)	0.0083	0.0331*
9. Do you prefer to stay at home, rather than going out and doing new things?	0.257 (0.6121)	0.0001	6.418* (0.0113)	0.0032	0.0150*
15. Do you think that most people are better off than you are?	15.138* (0.0000)	0.0185	15.138* (0.0000)	0.0194	0.0092

χ^2^: the value of the difference in −2 log-likelihood of models with and without group variable G for uniform DIF and models with and without interaction θG for non-uniform DIF.

Δβ_1_: Crane van Belle and Larson criterion or |[β_1_ (Model_without G_) - β_1_ (Model_with G_)]/β_1_ (Model_without G_)|.

ΔR_1_ = 1-ln[L(Model_with G_)]/ln[L(Model_without G_)]; ΔR_2_ = 1-ln[L(Model_with θG_)]/ln[L(Model_without θG_)].

* Represent the items showing uniform or non-uniform DIF;

P: p-value of Chi-square statistic.

**Table 4:** The results of comparing group differences with and without uniform DIF items.

		Total score		Total score corrected for DIF	
			
		Mean (SD^k^)	P-value^l^	Mean (SD^k^)	P-value^l^
Human settlements^a^	Village	5.51 (4.00)	0.010^j^	2.60 (2.12)	0.001^j^
	City	4.97 (4.09)		2.25 (2.11)	
Disease^b^	With chronic illness	5.60 (4.02)	<0.001^j^	4.62 (3.33)	<0.001^j^
	Without chronic illness	4.24 (4.00)		3.54 (3.35)	
Employment status^c^	Yes	3.81 (3.84)	<0.001^j^	2.05 (2.16)	<0.001^j^
	No	5.56 (4.04)		2.86 (2.30)	
Marital status^d^	Married	4.54 (3.87)	<0.001^j^	3.33 (2.92)	<0.001^j^
	Single	7.35 (3.93)		5.39 (3.06)	
Age^e^	60–74	4.87 (3.96)	<0.001^j^	4.01 (3.28)	<0.001^j^
	75–90	6.20 (4.20)		5.20 (3.55)	
Gender^f^	Male	4.59 (4.07)	<0.001^j^	0.82 (1.22)	0.170
	Female	6.02 (3.90)		0.87 (1.21)	
Homebound^g^	Yes	7.69 (3.71)	<0.001^j^	2.33 (1.58)	<0.001^j^
	No	4.72 (3.95)		1.61 (1.54)	
Financial status^h^	Rich	3.91 (3.67)	<0.001^j^	0.32 (0.57)	0.035
	Make ends meet	5.29 (4.01)		0.38 (0.59)	
	Poor	6.60 (4.04)		0.40 (0.61)	
Ethnic^i^	Persian	4.54 (3.88)	<0.001^j^	3.41 (3.03)	<0.001^j^
	Non-Persian	5.79 (4.12)		4.16 (3.05)	

a Total score after removing uniform DIF items 1, 2, 5, 7, 8, 9, and 15.

b Total score after removing uniform DIF items 3, 4, and 10.

c Total score after removing uniform DIF items 1, 2, 4, 8, 9, 11, and 15.

d Total score after removing uniform DIF items 5, 9, 11, and 15.

e Total score after removing uniform DIF items 5 and 7.

F Total score after removing uniform DIF items 2, 4, 5, 6, 7, 8, 9, 13, 14, and 15.

g Total score after removing uniform DIF items 1, 2, 4, 6, 8, 9, 10, 12,14, and 15.

h Total score after removing uniform DIF items 1, 2, 3, 4, 5, 6, 7, 8, 9, 12, 13, 14, and 15.

i Total score after removing uniform DIF items 1, 8, 9, and 15.

j Represent the significant association to α = 0.01 with or without DIF items.

k Standard deviation.

l P-value of Mann-Whitney U test.

## Footnotes

1 Δβ_1=_|[β_1_ (Model_without G_) - β_1_ (Model_with G_)]/β_1_ (Model_without G_)|.

2 ΔR_1_ = 1-ln[L(Model_with G_)]/ln[L(Model_without G_)].

3 ΔR_2_ = 1-ln[L(Model_with θG_)]/ln[L(Model_without θG_)].
